# Gemcitabine-based chemotherapy as a viable option for treatment of advanced breast cancer patients: a meta-analysis and literature review

**DOI:** 10.18632/oncotarget.23426

**Published:** 2017-12-19

**Authors:** Zhibo Xie, Yifan Zhang, Chen Jin, Deliang Fu

**Affiliations:** ^1^ Department of Pancreatic Surgery, Pancreatic Disease Institute, Huashan Hospital, Shanghai Medical College, Fudan University, Shanghai 200040, China; ^2^ Department of Plastic and Reconstructive Surgery, Shanghai Ninth People's Hospital, School of Medicine, Shanghai Jiao Tong University, Shanghai 200011, China

**Keywords:** gemcitabine, advanced breast cancer, chemotherapy, tumor response, adverse events

## Abstract

This meta-analysis was designed to compare the efficacy and safety of gemcitabine-based regimens for the treatment advanced breast cancer (ABC). Altogether 15 studies involving 8195 ABC patients were retrieved for analysis. Compared with non-gemcitabine-based chemotherapies, patients receiving gemcitabine-based therapy exhibited better overall survival (OS), progression free survival (PFS), and objective response rate (ORR) (HR = 1.12, 95% CI 1.05 to 1.19; HR = 1.16, 95% CI 1.03 to 1.30; HR = 1.14, 95% CI 1.04 to 1.24). Grade 3/4 hematologic toxicity was significantly high but manageable in gemcitabine-based groups. Subgroup analysis revealed that patients with first-line gemcitabine-based chemotherapy had better OS (HR = 1.19, 95% CI 1.07 to 1.32), PFS (HR = 1.17, 95% CI 1.08 to 1.27), and ORR (RR = 1.16, 95% CI 1.02 to 1.32). In addition, additional gemcitabine chemotherapy also showed better OS (HR = 1.17, 95% CI 1.06 to 1.30), PFS (HR = 1.20, 95% CI 1.11 to 1.30) and ORR (RR = 1.23, 95% CI 1.06 to 1.42) than gemcitabine replacement therapy. Furthermore, patients receiving gemcitabine-taxanes-based regimens had better OS (HR = 1.17, 95% CI 1.06 to 1.28), PFS (HR = 1.12, 95% CI 1.04 to 1.20) and ORR (RR = 1.17, 95% CI 1.01 to 1.35) than patients with non-gemcitabine-taxanes-based chemotherapy. These findings indicate that gemcitabine combination regimens could serve as a promising regimen for ABC patients, though increased hematologic toxicity should be considered with caution.

## INTRODUCTION

Breast cancer is one of the most prevalent cancers, causing approximately half a million deaths per year worldwide [1]. Among women with breast cancer in Western countries, 30%–40% had advanced breast cancer (ABC) [2], in whom the median survival is 2–3 years, and the 5-year survival is 5–10% [3]. The principal goal of current therapies for the treatment of ABC patients is to seek a longer progression-free survival (PFS) and better symptom relief without increasing toxicity or compromising the quality of life (QoL) [4].

Among the chemotherapeutic agents currently available for the treatment of ABC, anthracyclines and taxanes are considered the most active and represent widely used treatment options [5, 6]. However, ABC is likely to progress due to primary or acquired resistance to these chemotherapy drugs. In addition, chemotherapy regimens containing anthracyclines and/or taxanes are now often considered as the standard option for adjuvant treatment of early breast cancer, especially in high-risk women, thus limiting their use in patients who subsequently develop disease relapse [7, 8].

New cytotoxic treatments for disseminated disease, such as gemcitabine, capecitabine, and vinorelbine, are now available for patients who have been previously treated with anthracyclines and taxanes [9–11]. Gemcitabine (Gemzar; 2′, 2′-difluorodeoxycytidine) is a pyrimidine antimetabolite and a specific analogue of deoxycytidine widely used in many kinds of solid tumors or neoplastic hematologic disorders. In ABC patients, the clinical outcomes of trials evaluating combination regimens containing gemcitabine are inconsistent. Many clinical trials with large sample size discovered that combination regimens containing gemcitabine could prolong both overall survival (OS) and PFS without increasing the occurrence of serious adverse events (ADEs) [12, 13]. Other medical centers argued that OS and PFS were similar between patients with or without gemcitabine regimens, and that gemcitabine was also associated with serious ADEs in individual cases [14]. The above results show that there are controversies about the benefit of gemcitabine-based combination regimens. In addition, some studies used gemcitabine as the first-line treatment [15], while studies used it as the second-line treatment in ABC patients who were pretreated with anthracycline- or taxane [16]. What's more, chemotherapy regimens for ABC patients are multifarious in different studies. In some studies, the chemotherapy regimen was used as a control group, and gemcitabine plus the same chemotherapy regimen as the experimental group [12, 17, 18]. In other studies, researchers used gemcitabine combination regimens as the experimental group, and another totally different chemotherapy regimen as the control group [19].

Given the above confusion, it is necessary to find out the significance of gemcitabine in ABC patients and testify whether gemcitabine-based combination regimens will bring more benefits to these patients. In the current study, we focused on gemcitabine-based chemotherapies versus non-gemcitabine-based therapies for the treatment of ABC.

## RESULTS

### Search strategy and selection criteria

**Figure 1:**
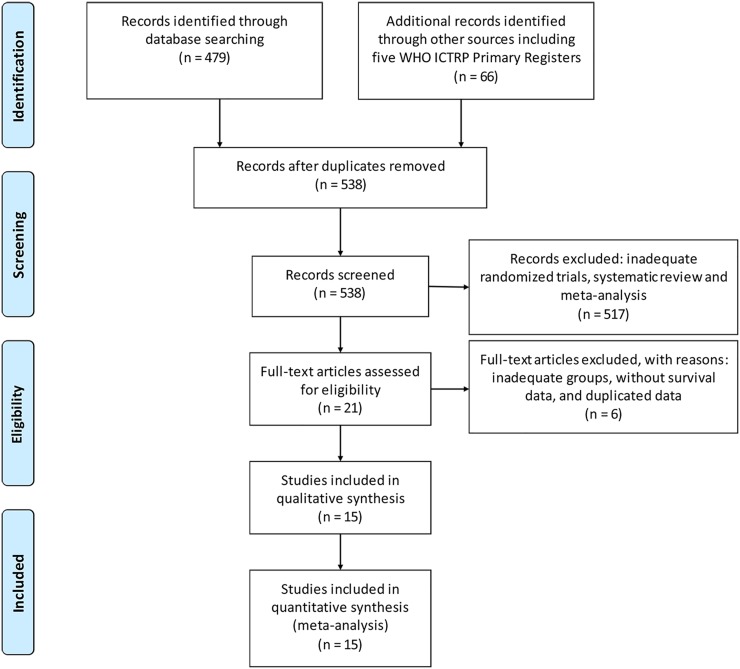
A flow diagram of the search strategy

Initial searching of literature databases and trial registries revealed 479 published clinical trials and 66 registered ones (Figure 1). After removing 7 duplicates, 538 potentially eligible trials were left, of which 517 trials were excluded after reviewing the abstracts because the study design or outcome data did not satisfy the inclusion criteria. After reading the full text of the remaining 21 trials, 6 studies [20–25] were further excluded. The study of Feher *et al.* [20] was excluded because they used gemcitabine monotherapy in the experimental group, knowing that we only included studies with combination regimens containing gemcitabine. In addition, they enrolled postmenopausal women (aged 60 or older), which may produce selection bias for our analysis. The study of Park *et al.* [22] was also excluded because their patients were randomized to receive a combination of gemcitabine and vinorelbine or gemcitabine until disease progression followed by vinorelbine monotherapy. In Tomova *et al.*'s study [25], the patients were randomized to receive gemcitabine plus docetaxel administered intravenously for a total of eight cycles or four cycles of docetaxel followed by four cycles of gemcitabine. In Seidman *et al.*'s study [23], a crossover design was used. In above 3 studies [22, 23, 25], gemcitabine was used in both experimental and control groups, which was not eligible for our analysis. In Moinpour *et al.*'s study [21], survival data were not available. In another study published in 2014 by Seidman *et al.* [24], a pooled result was from Chan *et al*.'s study [26] and Seidman *et al.*'s study [23]. For these reasons, we excluded above six studies and enrolled the rest 15 studies [12–18, 26–33] in our systematic review (The Selection flow was is shown in Figure 1).

Altogether 15 studies [12–18, 26–33] with 8195 ABC patients were finally enrolled in our analysis (Table 1). Of them, 12 studies [12–18, 26, 29–31, 33] had 2-arm design, and the remaining 3 studies [27, 28, 32] had 3 arms design. In addition, five studies [16, 26, 29–31] used gemcitabine as second-line chemotherapy, and the others used gemcitabine as first-line chemotherapy. Nevertheless, seven studies [12, 13, 15, 17, 18, 29, 31] used additional gemcitabine chemotherapy. With respect to different chemotherapy regimens in different studies, the gemcitabine combined taxanes chemotherapy regimen was most commonly used in nine studies [12, 13, 15, 17, 18, 26, 31–33] of the 15 included studies.

**Table 1:** Baseline characteristics of included studies

Study	Arm	Patients, *n*	Regimens
Albain *et al.* 2008	Gem/Pac	266	Gem 1250 mg/m^2^ i.v. d_1,8_, Pac 175 mg/m^2^ i.v. d_1_, q21d
	Pac	263	Pac 175 mg/m^2^ i.v. d_1_, q21d
Amadori *et al.* 2013	Gem/Nvb	61	Gem 1200 mg/m^2^ i.v. d_1,8_, Nvb 30 mg/m^2^ i.v. d_1,8_, q21d
	Pem/Cbp	64	Pem 600 mg/m^2^ i.v. d_1_, Cbp an AUC of 5 i.v. d_1_, q21d
Brufsky *et al.* 2011	Gem/Pac/Beva	93	Pac 90 mg/m^2^ i.v. d_1,8,15_, Beva 10 mg/kg i.v. d_1,15_, Gem 1500mg, d_1,15_,q28d
	Pac/Beva	94	Pac 90 mg/m^2^ i.v. d_1,8,15_, Beva 10 mg/kg i.v. d_1,15_, q28d
Chan *et al.* 2009	Gem/Doc	152	Gem 1000 mg/m^2^ i.v. d_1,8_, Doc 75 mg/m^2^ i.v. d_1_, q21d
	Cap/Doc	150	Cap 1250 mg/m^2^ bid d_1-14_, Doc 75 mg/m^2^ i.v. d_1_, q21d
Fountzilas *et al.* 2009	Pac/Cbp	136	Pac 175 mg/m^2^ i.v. d_1_, Cbp an AUC of 6 d_1_, q3w for 6 cycles
	Gem/Doc	134	Gem 1000 mg/m^2^ i.v. d_1,8_, Doc 75 mg/m^2^ i.v. d_8_, q3w for 6 cycles
	Pac	136	Pac 80 mg/m^2^ i.v., q1w for 12 cycles
Gómez *et al.* 2016	Lap/Cap	51	Lap 1250 mg p.o. d_1-14_, Cap 2000 mg/m^2^ p.o. d_1-14_
	Lap/ Nvb	45	Lap 1250 mg p.o. d_1-14_, Nvb 25 mg/m^2^ i.v. d_1, 8_
	Lap/Gem	46	Lap 1250 mg p.o. d_1-14_, Gem 1000 mg/m^2^ d_1, 8_
Joensuu *et al.* 2010	Doc	115	Doc 100 mg/m^2^ i.v.d_1_, q21d
	Doc/Gem	122	Doc 100 mg/m^2^ i.v.d_1_, q21d, Gem 1000 mg/m^2^ i.v. d_1,8,_q21d
Martín *et al.* 2007	Gem/Nvb	125	Nvb 30 mg/m^2^ i.v. d_1,8,_ Gem 1200 mg/m^2^ i.v. d_1,8_, q21d
	Nvb	126	Nvb 30 mg/m^2^ i.v. d_1,8_, q21d
Nielsen *et al.* 2011	Gem/Doc	170	Gem 1200 mg/m^2^ i.v. d_1,8_, Doc 75 mg/m^2^ i.v. d_8,_ q21d
	Doc	167	Doc 100 mg/m^2^ i.v. d_1_, q21d
Pallis *et al.* 2011	Gem/Nvb	74	Nvb 25 mg/m^2^ i.v. d_1,15_, Gem 1000 mg/m^2^ i.v. d_1,15_ ,q28d for 6 cycles
	Cap	74	Cap 1250 mg/m^2^ bid, d_1-14_, q21d for 6 cycles
Papadimitriou *et al.* 2009	Doc/Gem	41	Doc 35 mg/m^2^ i.v. d_1,8,15_, Gem 600 mg/m^2^ i.v. d_1,8,15_, q3w for 6 cycles
	Doc	34	Doc 40 mg/m^2^ i.v. d_1,8,15_, q3w for 6 cycles
Park *et al.* 2013	Gem/Pac → Gem/Pac	116	Gem 1250 mg/m^2^ i.v. d_1,8_, Pac 175 mg/m^2^ i.v. d_1_, q21d
	Gem/Pac → Obersavation	115	/
Swain *et al.* 2013	Doc/Dox/Cyc	1630	Dox 50 mg/m^2^ i.v., Cyc 500 mg/m^2^ i.v., Doc 75 mg/m^2^ i.v. q3w
	Dox/Cyc → Pac	1634	Dox 60 mg/m^2^ i.v., Cyc 600 mg/m^2^ i.v., q2w for 4 cycles → Pac 175 mg/m^2^ i.v. q2w for 4 cycles
	Dox/Cyc → Gem/Pac	1630	Dox 60 mg/m^2^ i.v., Cyc 600 mg/m^2^ i.v., q2w for 4 cycles → Pac 175 mg/m^2^ i.v., Gem 2000 mg/m^2^ i.v.q2w for 4 cycles
Vici *et al.* 2011	Gem/Doc	36	Gem 1000 mg/m^2^ i.v. d_1,8_, Doc 75 mg/m^2^ i.v. d_1_, q21d for 8 cycles
	Cap/Doc	36	Cap 1250 mg/m^2^ bid d_1-14_, Doc 75 mg/m^2^ i.v. d_1_, q21d for 8 cycles
Zielinski *et al.* 2005	Gem/Epi/Pac	124	Gem 1000 mg/m^2^ i.v. d_1,4_,Epi 90 mg/m^2^ i.v. d_1_, Pac 175 mg/m^2^ i.v. d_1_, q21d
	5-FU/Epi/Ctx	135	5-FU 500 mg/m^2^ i.v. d_1_, Epi 90 mg/m^2^ i.v. d_1_,Ctx 500 mg/m^2^ d_1_, q21d

*Abbreviation*: Beva = bevacizumab, Cap = capecitabine, Cbp = carboplatin, Cyc = cyclophosphamide, Doc = docetaxel, Dox = doxorubicin, Epi = epirubicin, Gem = gemcitabine, Lap = lapatinib, Nvb = vinorelbine, Pac = paclitaxel, Pem = pemetrexed, 5-Fu = 5-fluorouracil.

### Survival and tumor response

In the three studies with 3-arm design [27, 28, 32], survival data were presented as survival curves, and therefore we only included studies with 2-arm design for survival analysis. But as these three studies provided objective response rate (ORR) data, they were eligible and included for ORR analysis.

### Overall survival

**Figure 2:**
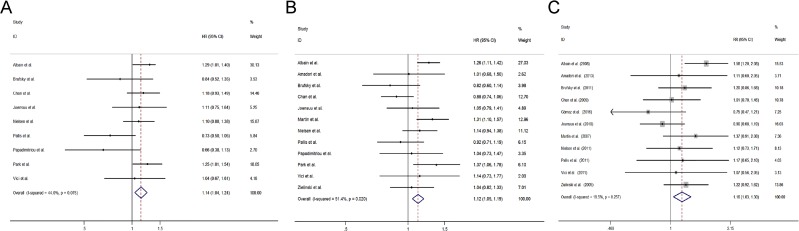
Comparisons of primary outcomes between gemcitabine-containing and non–gemcitabine-containing regimens (**A**) Overall survival, (**B**) Progression free survival, (**C**) Objective tumor response rate.

We first conducted meta-analysis about OS and found that nine studies [12–18, 26, 29–31, 33] with two-arm design evaluated OS. Pooled results showed that combination regimens containing gemcitabine significantly prolonged the OS of ABC patients [hazard ratio (HR) = 1.14, 95% confidence interval (CI) 1.04 to 1.24)] (Figure 2A).

Moreover, the OS data were also reported in studies with 3-arm design. In Fountzilas *et al.*'s study [27], the median survival was 29.9 months in patients using paclitaxel and carboplatin, 26.9 months in patients using docetaxel and gemcitabine, and 41.0 months in patients using monotherapy of paclitaxel (*P* = 0.037). Nevertheless, Swain *et al.* [32] also presented the OS data. They randomized patients to receive six cycles of docetaxel (T), doxorubicin (A), and cyclophosphamide (C) (TAC), four cycles of dose-dense (DD) doxorubicin and cyclophosphamide followed by four cycles of DD paclitaxel (P) (AC→P), or DD AC→P with four cycles of gemcitabine (G) added to the DD paclitaxel (DD AC→PG)and found that OS was similar between patients with AC→PG and patients with AC→P (HR = 0.85, *P* = 0.130) or between patients with AC→PG and patients with TAC (HR = 0.86, *P* = 0.170). They showed that combination regimens containing gemcitabine did not bring significant benefits to ABC patients in terms of OS.

### Progression free survival

Pooled results from 12 studies with two-arm design [12–18, 26, 29–31, 33] revealed that patients received combination regimens containing gemcitabine had a significant better PFS (HR = 1.12, 95% CI 1.05 to 1.19) (Figure 2B).

In studies with 3-arm design, Fountzilas *et al.* [27] found that PFS was similar between their 3 groups (mean PFS: 11.5 months for paclitaxel and carboplatin; 10.4 months for docetaxel and gemcitabine; 11.4 months for paclitaxel; *P* = 0.570). Similarly, Gómez *et al.* [28] found that there was no significant difference in PFS between patients in three arms (mean PFS: 9.1 months for lapatinib and capecitabine; 7.0 months for lapatinib and vinorelbine; 6.8 months for lapatinib and gemcitabine; *P* = 0.476). Swain *et al.* [32] also showed a similar PFS between patients in three arms (AC→PG *vs.* TAC, HR = 0.93, *P* = 0.390; AC→PG *vs.* AC→P, HR = 1.07, *P* = 0.410).

### Objective response rate

Altogether 11 studies including 10 studies with two-arm design [12–18, 26, 29, 30, 33] and 1 study with three-arm design [28] presented the outcome of ORR and found that patients receiving combination regimens containing gemcitabine had a significant better ORR (risk ratio (RR) =1.16, 95% CI 1.03 to 1.30) (Figure 2C).

### Toxicity

We carefully reviewed all the included studies in our analysis, and found that these studies included over 50 different ADEs. Among them, we provided nine most common ADEs and compared the incidence of grade 3 or 4 ADEs between patients receiving chemotherapy regimens with or without gemcitabine.

**Figure 3:**
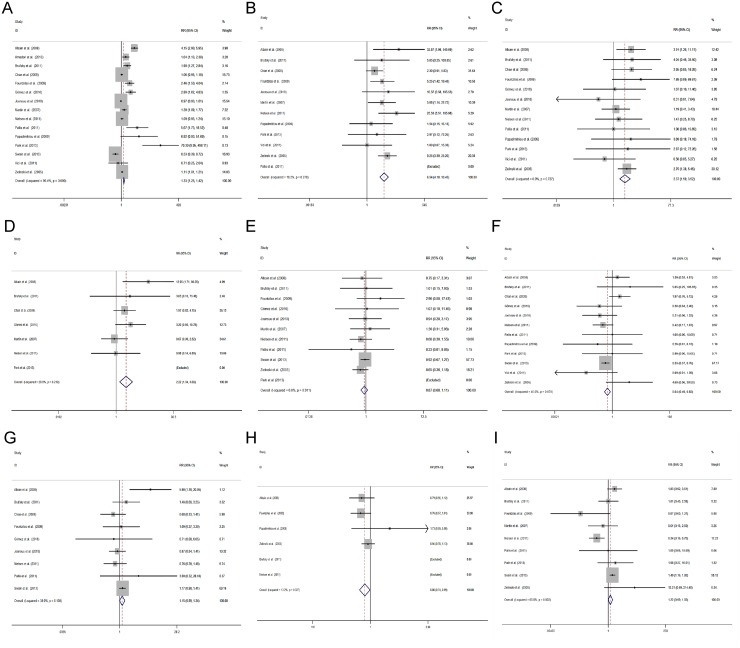
Comparisons of toxicity between gemcitabine-containing and non–gemcitabine-containing regimens (**A**) Neutropenia, (**B**) Thrombocytopenia, (**C**) Anemia, (**D**) Aalanine aminotransferase, (**E**) Nausea/vomiting, (**F**) Diarrhea, (**G**) Fatigue, (**H**) Alopecia, (**I**) Neuropathy.

It was found that patients receiving combination regimens containing gemcitabine were more likely to have a higher incidence of neutropenia (RR = 1.33, 95% CI 1.25 to 1.42) (Figure 3A), thrombocytopenia (RR = 6.54, 95% CI 4.10 to 10.45) (Figure 3B), anemia (RR = 2.37, 95% CI 1.59 to 3.52) (Figure 3C), and an increased level of serum alanine aminotransferase (ALT) (RR = 2.22, 95% CI 1.34 to 3.69) (Figure 3D). In addition, patients receiving combination regimens containing gemcitabine were more likely to have less severe diarrhea (RR = 0.64, 95% CI 0.49 to 0.83) (Figure 3F), alopecia (RR = 0.86, 95% CI 0.74 to 0.99) (Figure 3H). The incidence of nausea/vomiting (RR = 0.87, 95% CI 0.68 to 1.11) (Figure 3E), fatigue (RR = 1.15, 95% CI 0.99 to 1.34) (Figure 3G), and neuropathy (RR = 1.22, 95% CI 0.99 to 1.50) was similar between 2 groups (Figure 3I).

### Quality of life

Altogether 3 studies [16, 17, 27] reported the outcome of QoL in ABC patients. Amadori *et al.* [16] and Fountzilas *et al.* [27], reported that there was no significant difference in QoL between the treatment groups, while Brufsky *et al.* [17] found that QoL in patients with non-gemcitabine chemotherapy was better improved than that in patients with gemcitabine chemotherapy. According to our initial literature research, Moinpour *et al.* [21] found that QoL comparison study favored the gemcitabine combination therapy.

### Subgroup analysis

### Comparision between gemcitabine-containing and non–gemcitabine-containing regimens in first-line setting

**Figure 4:**
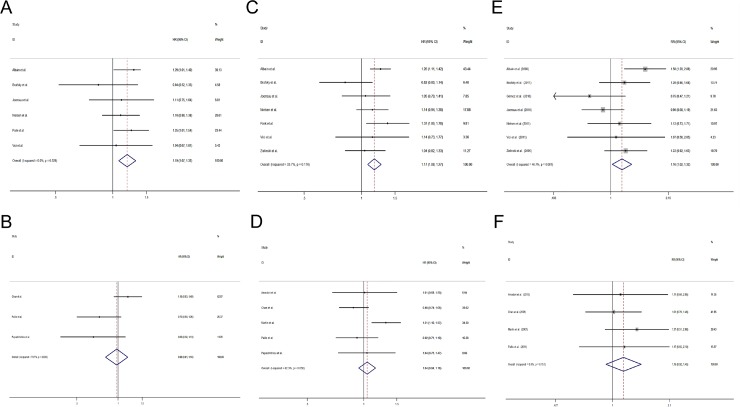
Comparisons of primary outcomes between studies using gemcitabine-containing regimens as first-line and second-line treatment (**A**) Overall survival (first-line), (**B**) Overall survival (second-line) (**C**) Progression free survival (first-line), (**D**) Progression free survival (second-line), (**E**) Objective tumor response rate (first-line), (**F**) Objective tumor response rate (second-line).

Altogether 10 studies used gemcitabine as the first-line treatment [12–15, 17, 18, 27, 28, 32, 33] and the other 5 studies [19, 26, 29–31] used gemcitabine as the second-line chemotherapy. In studies using gemcitabine as first-line treatment, patients with gemcitabine-containing regimens had a significantly longer OS (HR = 1.19, 95% CI 1.07 to 1.32) (Figure 4A), PFS (HR = 1.17, 95% CI 1.08 to 1.27) (Figure 4C) and a higher ORR (RR = 1.16, 95% CI 1.02 to 1.32) (Figure 4E) than patients with non-gemcitabine-containing regimens. However, the trend is not seen in studies using gemcitabine as post first-line treatment (OS: HR = 0.98, 95% CI 0.81 to 1.18, Figure 4B; PFS: HR = 1.04, 95% CI 0.94 to 1.16, Figure 4D; ORR: RR = 1.15, 95% CI 0.92 to 1.45, Figure 4F).

### Comparison of the efficacy between additional gemcitabine and gemcitabine replacement regimens

**Figure 5:**
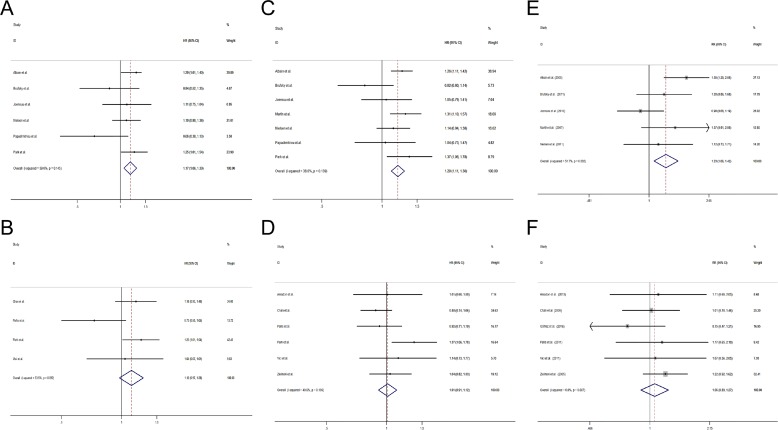
Comparisons of primary outcomes between studies using additional gemcitabine and studies using gemcitabine as replacement chemotherapy (**A**) Overall survival (additional gemcitabine chemotherapy), (**B**) Overall survival (gemcitabine replacement chemotherapy) (**C**) Progression free survival (additional gemcitabine chemotherapy), (**D**) Progression free survival (gemcitabine replacement chemotherapy), (**E**) Objective tumor response rate (additional gemcitabine chemotherapy), (**F**) Objective tumor response rate (gemcitabine replacement chemotherapy).

Of the 15 studies in our analysis, 7 studies used additional gemcitabine chemotherapy [12, 15, 17, 18, 29–31], and the other 8 used gemcitabine replacement regimens. In studies using additional gemcitabine chemotherapy, a significantly longer OS (HR = 1.17, 95% CI 1.06 to 1.30) (Figure 5A), PFS (HR = 1.20, 95% CI 1.11 to 1.30) (Figure 5C) and a higher ORR (RR = 1.23, 95% CI 1.06 to 1.42) (Figure 5E) were found, while the difference in OS (HR = 1.12, 95% CI 0.97 to 1.28) (Figure 5B), PFS (HR = 1.01, 95% CI 0.91 to 1.12) (Figure 5D) and ORR (RR = 1.06, 95% CI 0.89 to 1.27) (Figure 5F) was similar in patients receiving gemcitabine replacement regimens.

### Efficacy of gemcitabine and taxanes combination therapy

**Figure 6:**
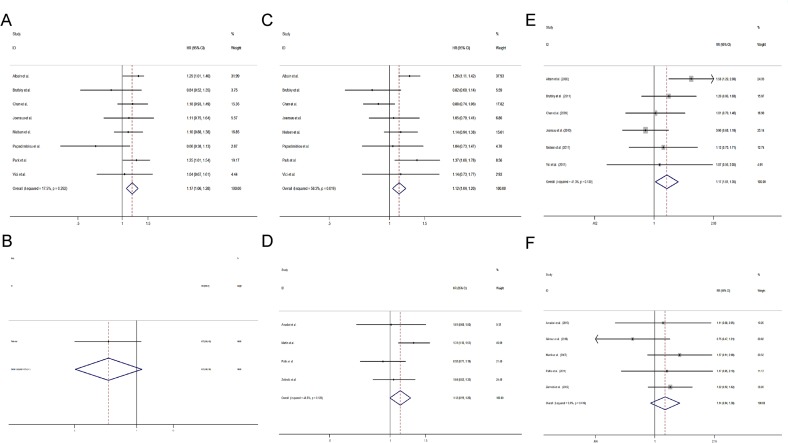
Comparisons of primary outcomes between studies with gemcitabine- taxanes-based regimens and studies with non-gemcitabine-taxanes-based regimens (**A**) Overall survival (gemcitabine-taxanes-based regimens), (**B**) Overall survival (non-gemcitabine-taxanes-based regimens) (**C**) Progression free survival (gemcitabine-taxanes-based regimens), (**D**) Progression free survival (non-gemcitabine-taxanes-based regimens), (**E**) Objective tumor response rate (gemcitabine-taxanes-based regimens), (**F**) Objective tumor response rate (non-gemcitabine-taxanes-based regimens).

Nine [12, 13, 15, 17, 18, 26, 31–33] of the 15 included studies used gemcitabine combined taxanes chemotherapy regimen. Pooled results of studies combining gemcitabine and taxanes showed that patients using the gemcitabine-containing regimens had a better OS (HR = 1.17, 95% CI 1.06 to 1.28) (Figure 6A), PFS (HR = 1.12, 95% CI 1.04 to 1.20) (Figure 6C) and ORR (RR = 1.17, 95% CI 1.01 to 1.35) (Figure 6E) than patients with non-gemcitabine-containing regimens. However, there was no significant difference in OS (HR = 0.78, 95% CI 0.60 to 1.26) (Figure 6B), PFS (HR = 1.12, 95% CI 0.99 to 1.26) (Figure 6D) and ORR (RR = 1.14, 95% CI 0.94 to 1.36) (Figure 6F) between studies using non-gemcitabine-taxanes-combination therapy.

During the initial literature searching process, we found that there was a meta-analysis comparing the safety and efficacy of gemcitabine and taxanes combination therapy in ABC patients [34]. They found that ORR and time to progression were superior for gemcitabine/taxanes-treated patients (ORR, odd ratio = 1.28, 95% CI 1.07 to 1.53; time to progression, HR = 0.80, 95% CI 0.71 to 0.89). And gemcitabine/ taxanes-based combination significantly improved OS in the first-line subgroup (HR = 0.84; 95% CI 0.71 to 0.99).

### Sensitivity analysis

To test whether our meta-analysis results were skewed because of the study with high risk of bias, we repeated the analyses after excluding the study [14] (the initial quality assessment of included studies is shown in Supplementary Table 1). The results for PFS (HR = 1.12, 95% CI 1.05 to 1.20) (Supplementary Figure 1) and ORR (RR = 1.15, 95% CI 1.02 to 1.30) (Supplementary Figure 2) were similar to those obtained when including the study with high risk of bias, suggesting that our meta-analysis is reliable.

Moreover, we also switched the fix- and random-model to test the sensitivity of the outcome, and found that recalculated ORR (RR = 1.14, 95% CI 1.00 to 1.30, random model) (Supplementary Figure 3) was similar to the previous ORR (RR = 1.16, 95% CI 1.03 to 1.30, fix model).

### Publication bias

Funnel plots were generated and analyzed using Egger's tests in order to assess the risk of publication bias in all included studies. The funnel plots for ORR appeared to be symmetrical, suggesting the absence of bias. This was corroborated by Egger's test (*t* = −0.77, *P* = 0.459) (Supplementary Figure 4).

## DISCUSSION

The treatment of ABC is a clinical challenge because of its propensity for late presentation with inoperable disease, aggressive tumor biology, and resistance to chemotherapy [35–37]. Taxane/anthracycline-based chemotherapy is the treatment of choice in most cancer centers and countries for the treatment of ABC, and a wide variety of single agents or combination therapies have been investigate for patients previously treated with both a taxane and an anthracycline [38]. As an outstanding anti-tumor agent, gemcitabine has been evaluated in many randomized controlled trials for ABC, showing a favorable clinical outcome [25, 29]. However, whether gemcitabine-containing chemotherapy is better than non–gemcitabine-containing treatment still lacks adequate evidence. To compare the efficacy and tolerability of gemcitabine-containing chemotherapy in the treatment of ABC patients with sufficient statistical power, we performed this systematic review to overcome the statistical limitations (for instance, low case load) of the individual trials by comparing the treatment efficacy, safety profile and survival benefit between various subgroups.

This systematic review revealed that gemcitabine-containing chemotherapy was associated with significantly enhanced OS (HR = 1.14, 95% CI 1.04 to 1.24), PFS (HR = 1.12, 95% CI 1.05 to 1.19), and ORR (RR = 1.16, 95% CI 1.03 to 1.30) over non–gemcitabine-containing chemotherapy. Our initial review revealed another meta-analysis that also conducted a similar comparison [39]. Interestingly, they reported that OS, PFS and ORR were similar between ABC patients with or without gemcitabine-containing regimens. In their study, 9 trials including 2651 patients [12, 14, 15, 17, 18, 20, 26, 29, 30] were eligible for the meta-analysis. Compared with their inclusion, we additionally include 7 studies, and excluded Feher *et al.*'s study [20] because they used monotherapy of gemcitabine in the experimental group and only included postmenopausal women aged 60 years or older, which we were afraid may produce significant selection bias in our analysis. According to Brufsky *et al.*'s study [17], gemcitabine-containing chemotherapy was associated with significantly improved ORR as compared with non-gemcitabine-containing chemotherapy in patients younger than 65 years. Nevertheless, the trend was not significant in patients older than 65 years, indicating that age may affect the survival outcome. Included patients with older age were often postmenopausal women, and estrogen deficiency may affect the efficacy of chemotherapy [40]. Li *et al.* [39] found a significant bias in Feher *et al.*'s study and therefore excluded it from their meta-analysis. Interestingly, they also found that the insignificant difference between 2 arms turned significant. The dramatic change demonstrated a selection bias and inconvincible results in Li *et al.*'s [39] study, and we excluded Feher *et al.*'s study and conducted a new systematic review in order to provide more authentic results.

Severe toxicity is an important concern when combination chemotherapy is considered, especially in elderly patients and those who have poor PS score. Thus, toxicity and QoL have become equally important in assessing the therapeutic efficacy and determining the course of palliative chemotherapy. Toxicity is a major concern with gemcitabine. It was found in our study that additional use of gemcitabine increased the incidence of grade 3–4 hematopoietic system toxicity including neutropenia, thrombocytopenia and anemia, and increased the serum level of ALT [41]. Although gemcitabine combining regimens significantly increased the incidence of grade 3–4 hematopoietic and liver function impairment, few serious AEDs were reported. According to Zielinski *et al.* [14] thrombocytopenia did not constitute a clinical problem in patients treated with gemcitabine-combining regimens, because no bleeding episodes was reported. The rate of grade 3 and 4 myelosuppressive activity in patients with second-line therapy was lower as compared with first-line treatment studies with similar toxicity profiles, which could be attributed to the difference in the total durations of drug exposure in the previous treatment [42]. Furthermore, grade 3 or 4 hematologic toxicities were not commonly associated with clinical events such as transfusions [20]. Viewing the ADEs by age, some differences in toxicities would be noted. In patients with elder age, the severity and the incidence may increase [20]. Thus the toxic effects of the gemcitabine combination regimens are manageable when they are used with caution in elderly patients. QoL difference between patients with or without gemcitabine containing chemotherapy varies greatly, and further investigation is required before a convincing conclusion can be drawn.

Subgroup analysis of first- or second-line therapy revealed that gemcitabine containing chemotherapy was beneficial to ABC patients when it was used as the first-line therapy. Interestingly, gemcitabine has shown a modest activity as a single agent in ABC, especially when the drug was used as second-line or third-line treatment [43]. The satisfied survival benefit may be attributed to the included populations. Gemcitabine containing chemotherapy was used as the second-line therapy in these patients, mainly because of drug resistance to the previous first-line therapy or disease progression. Indeed, any therapy may not be as effective as expected in these patients. Thus, the result of survival benefits of gemcitabine containing regimens in first-line treatment would help clinical doctors make rational and efficacious chemotherapeutic protocols. According to Carrick *et al.* [44], combination regimens had a higher response rate than single-drug regimens, but sequential use of a single agent was associated with better QoL and similar or nearly similar survival, which may be the reason why mono-chemotherapy has widely been accepted as a better approach to breast cancer management. Nevertheless, the above-mentioned results were not based on gemcitabine containing regimens. Subgroup analysis in our study showed that gemcitabine, when used as an additional agent in chemo-regimens, brought significant survival and tumor response benefits as compared with gemcitabine replacement chemotherapy. In addition, a large multicenter trial reported that the combination was more effective than single-agent paclitaxel in terms of the response rate, time-to-disease progression, and OS [12] suggesting that gemcitabine combining regimens could bring better survival outcomes and tumor response. In our study, gemcitabine containing regimens increased the incidence of hematologic toxicity, though it was manageable.

In addition, QoL comparison in some studies favored the gemcitabine combination therapy. However, we did not compare the difference in survival and tumor response between the gemcitabine combination regimen and the single gemcitabine regimen. Further study is required to compare the efficacy of single gemcitabine in ABC patients. It was found in our study that gemcitabine combined with taxanes was the most commonly used regimen. Currently, taxanes are usually introduced early in the treatment of ABC patients’ treatment. In addition, taxanes are commonly used in patients with no or minimal prior anthracycline exposure and/or in combination with anthracyclines and gemcitabin [45]. Gemcitabine combined with taxanes doublet is a well-tolerated choice for ABC women after adjuvant anthracycline therapy or in whom the cardiotoxic effects of anthracyclines preclude its use [12]. Despite the manageable toxicity, the analysis of the global QoL end point from the QoL companion study still favored the regimens containing gemcitabine and taxanes [21]. A recently published meta-analysis compared the efficacy and toxicity in patients receiving chemotherapy with or without gemcitabine-taxanes-based regimens [34], and found that ORR and time to progression were superior to gemcitabine and taxanes treated patients, which is consistent with our subgroup analysis. They also found that gemcitabine-taxanes-based combination also significantly improved OS in the first-line subgroup (HR = 0.84; 95% CI 0.71 to 0.99). All evidence suggests that gemcitabine-taxanes-based chemotherapy may be a feasible regimen for ABC patients.

The biggest limitation of this systematic review is the heterogeneity of the included studies. The study designs and study inclusion criteria are totally different between different studies, and the pooled results might have biases. In order to make our results more convincible, we conducted sensitivity analysis. By excluding the study with high risk of bias, the final results for PFS and ORR are similar to previous results, suggesting that our meta-analyses are reliable. In addition, we also switched the fix- and random-model to test the sensitivity of the outcome, and found that recalculated ORR (RR = 1.14, 95% CI 1.00 to 1.30, random model) were similar to previous ORR (RR = 1.16, 95% CI 1.03 to 1.30, fix model). Additionally, funnel plots of publication bias for ORR appear to be symmetrical, suggesting the absence of bias. All above evidence shows that the results in our study are convincible.

In conclusion, a gemcitabine-based regimen possesses meaningful anti-tumor activity in the treatment of ABC, repeatedly demonstrating outcomes favored to non–gemcitabine-containing agents, especially for first-line treatment in ABC patients. Gemcitabine-based regimens could serve as promising regimens, although increased hematologic toxicity should be considered.

## METHODS

This meta-analysis was conducted according to PRISMA guidelines (Checklist S1).

### Literature search strategy

Systematic searches of the following electronic databases were conducted through January 2017 without language restrictions: MEDLINE, EMBASE, the Cochrane Library, and the Chinese National Knowledge Infrastructure. We also searched five primary clinical trial registries recognized by the WHO International Clinical Trial Registry Platform: Australia and New Zealand Clinical Trial Registry (www.anzctr.org.au/), Chinese Clinical Trial Register (www.chictr.org.cn), ISRCTN (www.controlled-trials.com/isrctn/), U.S. National Institutes of Health Clinical Trials Database (www.clinicaltrials.gov/), and Clinical Trials Registry-India (www.ctri.in:8080/Clinicaltrials/index.jsp) [46, 47]. Eligible studies were identified using any of the following index words: “gemcitabine,” “chemotherapy,” and “breast cancer”. Relevant reviews and meta-analyses comparing combination regimens with or without gemcitabine for ABC were examined manually to identify additional eligible studies.

### Inclusion criteria

Studies included in our analysis had to satisfy the following criteria: (1) randomized clinical trials; (2) trials including a group that received combination regimens containing gemcitabine and a group that received chemotherapy regimens without gemcitabine; (3) trials reporting data on OS or PFS as the clinical outcome; (4) trials including ABC with histologically confirmed invasive breast cancer and measurable or non-measurable distant metastases as confirmed by histology and/or radiology); (5) trials reporting sufficient data to allow calculation of the RR or HR with 95% CI.

### Types of outcome measures

Primary outcomes evaluated in the meta-analysis were OS, PFS and tumor response. Tumor response was classified according to the Modified Response Evaluation Criteria in Solid Tumors (mRECIST) [48, 49]. Tumor responses were classified as complete response, partial response, stable disease and progressive disease. The ORR was calculated by summing the complete response rate and partial response rate. Secondary outcomes were toxicity and QoL. Toxicity was graded according to the National Cancer Institute Common Toxicity Criteria (Version 3.0). We only included grade 3 or 4 common ADEs into our analysis.

### Data extraction

The following data were extracted independently by two reviewers (Z.B.X and Y.F.Z): the first author's name, year of publication, tumor characteristics, number of patients, and line of therapy. For each group, the intervention, number of patients, ORR, duration of response, OS, PFS and toxicity were also collected. Any disagreements about study eligibility or extracted data were arbitrated by a third reviewer (D.L.F).

### Quality assessment

Two reviewers (Z.B.X and Y.F.Z) independently evaluated all the included trials and independently assessed the risk of bias for each study using criteria specified by Cochrane Collaboration Back Review Group [50].

### Statistical analysis

All statistical calculations were performed using Stata 12.0 (Stata Corp, College Station, TX, USA). Mantel-Haenszel RRs with corresponding 95% CIs were calculated for tumor response. Survival data (OS and PFS) were extracted from survival curves [51] and HRs with 95% CIs were calculated. The meta-analysis was carried out on an ‘intention-to-treat’ basis. Heterogeneity was assessed by calculating *I*^2^. When *I*^2^ was less than 50%, a fixed-effects model was used; when *I*^2^ was more than 50%, a random-effects model was used. Homogeneity between trials was assessed using the χ^2^ test with the significance threshold set at *P* > 0.1. To evaluate the robustness of the meta-analysis results, we repeated all meta-analyses using the other type of model (fixed- or random-effects); if both models gave the same meta-analysis results, we judged the result to be reliable. Publication bias was assessed using Egger's test and funnel plots [52, 53] in Stata 12.0.

## SUPPLEMENTARY MATERIALS FIGURES AND TABLES



## Abbreviations

ABC: advanced breast cancer
ADE: adverse event
CI: confidence interval
HR: hazard ratio
ORR: objective response rate
OS: overall survival
PFS: progression free survival
QoL: quality of life
RR: risk ratio
