# Supplementary material for: Gemcitabine-based chemotherapy as a viable option for treatment of advanced breast cancer patients: a meta-analysis and literature review
Source: Oncotarget. 2017 Dec 19;9(6):7148–61. doi: 10.18632/oncotarget.23426 (PMC5805543; doi:10.18632/oncotarget.23426)
Supplement: Supplementary file 1 [file oncotarget-09-7148-s001.pdf]

# Gemcitabine-based chemotherapy as a viable option for treatment of advanced breast cancer patients: a meta-analysis and literature review

## SUPPLEMENTARY MATERIALS

**Supplementary Table 1: Quality analysis of included studies**

| Index | No. 1 | No. 2 | No. 3 | No. 4 | No. 5 | No. 6 | No. 7 | No. 8 | No. 9 | No. 10 | No. 11 | No. 12 | No. 13 | No. 14 | No. 15 |
|---|---|---|---|---|---|---|---|---|---|---|---|---|---|---|---|
| Was the method of randomization adequate? | Ⓨ | Ⓨ | Ⓨ | Ⓨ | Ⓨ | Ⓨ | Ⓨ | Ⓨ | Ⓨ | Ⓨ | Ⓨ | Ⓨ | Ⓨ | Ⓨ | Ⓨ |
| Was the treatment allocation concealed? | Ⓤ | Ⓤ | Ⓨ | Ⓤ | Ⓤ | Ⓤ | Ⓨ | Ⓨ | Ⓤ | Ⓝ | Ⓝ | Ⓨ | Ⓝ | Ⓨ | Ⓨ |
| Were the groups similar at baseline regarding the most important prognostic indicators? | Ⓨ | Ⓤ | Ⓨ | Ⓨ | Ⓨ | Ⓤ | Ⓨ | Ⓨ | Ⓤ | Ⓨ | Ⓤ | Ⓨ | Ⓨ | Ⓨ | Ⓝ |
| Was the patient blinded to the intervention? | Ⓝ | Ⓝ | Ⓝ | Ⓝ | Ⓝ | Ⓝ | Ⓝ | Ⓝ | Ⓝ | Ⓝ | Ⓝ | Ⓝ | Ⓝ | Ⓝ | Ⓝ |
| Was the care provider blinded to the intervention? | Ⓝ | Ⓝ | Ⓝ | Ⓝ | Ⓝ | Ⓝ | Ⓝ | Ⓝ | Ⓝ | Ⓝ | Ⓝ | Ⓝ | Ⓝ | Ⓝ | Ⓝ |
| Was the outcome assessor blinded to the intervention? | Ⓝ | Ⓝ | Ⓝ | Ⓝ | Ⓝ | Ⓝ | Ⓝ | Ⓝ | Ⓝ | Ⓝ | Ⓝ | Ⓝ | Ⓝ | Ⓝ | Ⓝ |
| Were co-interventions avoided or similar? | Ⓨ | Ⓨ | Ⓨ | Ⓨ | Ⓨ | Ⓨ | Ⓨ | Ⓨ | Ⓨ | Ⓨ | Ⓨ | Ⓨ | Ⓨ | Ⓨ | Ⓨ |
| Was the compliance acceptable in all groups? | Ⓨ | Ⓨ | Ⓨ | Ⓨ | Ⓨ | Ⓨ | Ⓨ | Ⓨ | Ⓨ | Ⓨ | Ⓨ | Ⓨ | Ⓨ | Ⓨ | Ⓨ |
| Was the drop-out rate described and acceptable? | Ⓨ | Ⓨ | Ⓨ | Ⓨ | Ⓨ | Ⓨ | Ⓤ | Ⓨ | Ⓨ | Ⓨ | Ⓤ | Ⓨ | Ⓨ | Ⓨ | Ⓤ |
| Was the timing of the outcome assessment in all groups similar? | Ⓨ | Ⓨ | Ⓨ | Ⓨ | Ⓨ | Ⓨ | Ⓨ | Ⓨ | Ⓨ | Ⓨ | Ⓨ | Ⓨ | Ⓨ | Ⓨ | Ⓨ |
| Did the analysis include an intention-to-treat analysis? | Ⓨ | Ⓤ | Ⓨ | Ⓨ | Ⓨ | Ⓨ | Ⓤ | Ⓨ | Ⓨ | Ⓨ | Ⓤ | Ⓨ | Ⓨ | Ⓨ | Ⓝ |

Notes: Ⓨ = Yes; Ⓝ = No; Ⓤ = Unclear.
Study: No.1 = Albain *et al.*; No.2 = Amadori *et al.*; No.3 = Brufsky *et al.*; No.4 = Chan *et al.*; No.5 = Fountzilas *et al.*; No.6 = Gómez *et al.*; No.7 = Joensuu *et al.*; No.8 = Martín *et al.*; No.9 = Nielsen *et al.*; No.10 = Pallis *et al.*; No.11 = Papadimitriou *et al.*; No.12 = Park *et al.*; No.13 = Swain *et al.*; No.14 = Vici *et al.*; No.15 = Zielinski *et al.*

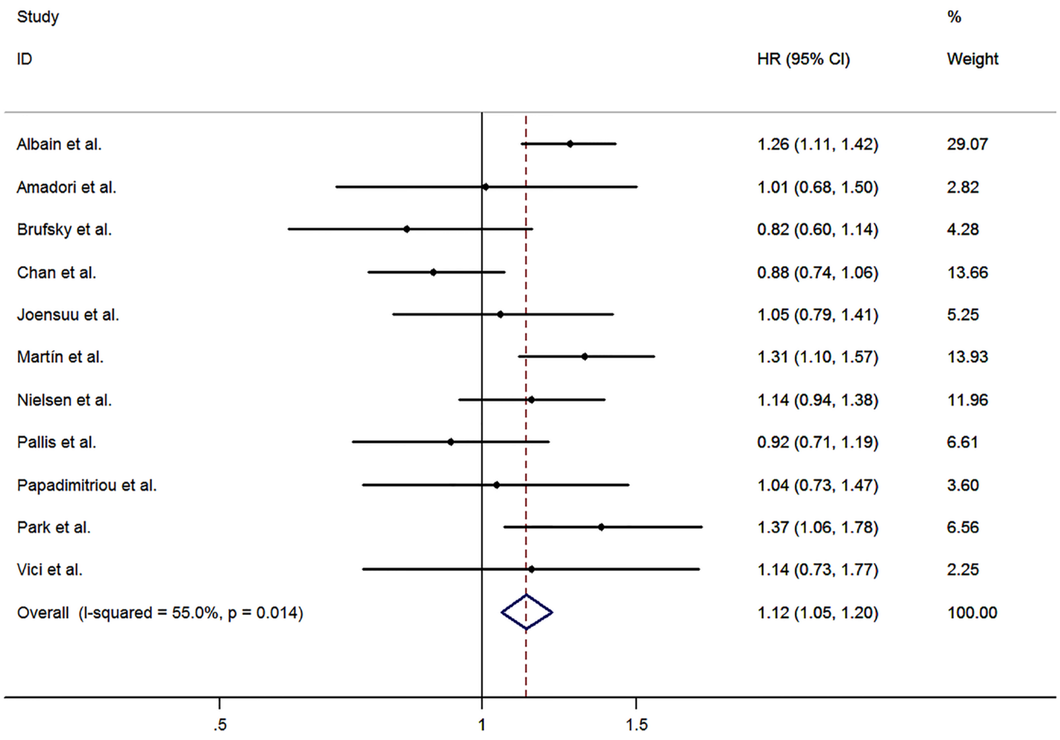


**Supplementary Figure 1: Comparisons of progression free survivals between 2 arms after excluding the study with the high risk of bias (Zielinski *et al.*).**

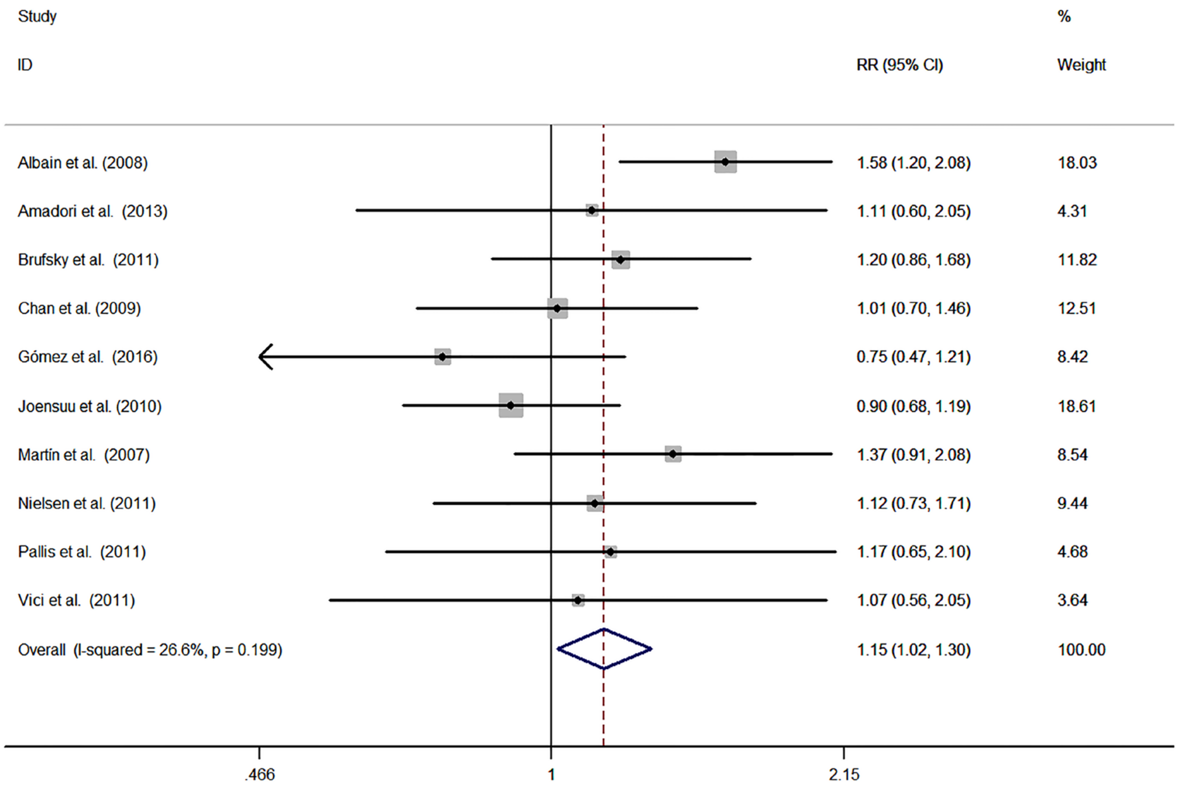


**Supplementary Figure 2: Comparisons of objective tumor response rate between 2 arms after excluding the study with the high risk of bias (Zielinski *et al.*).**

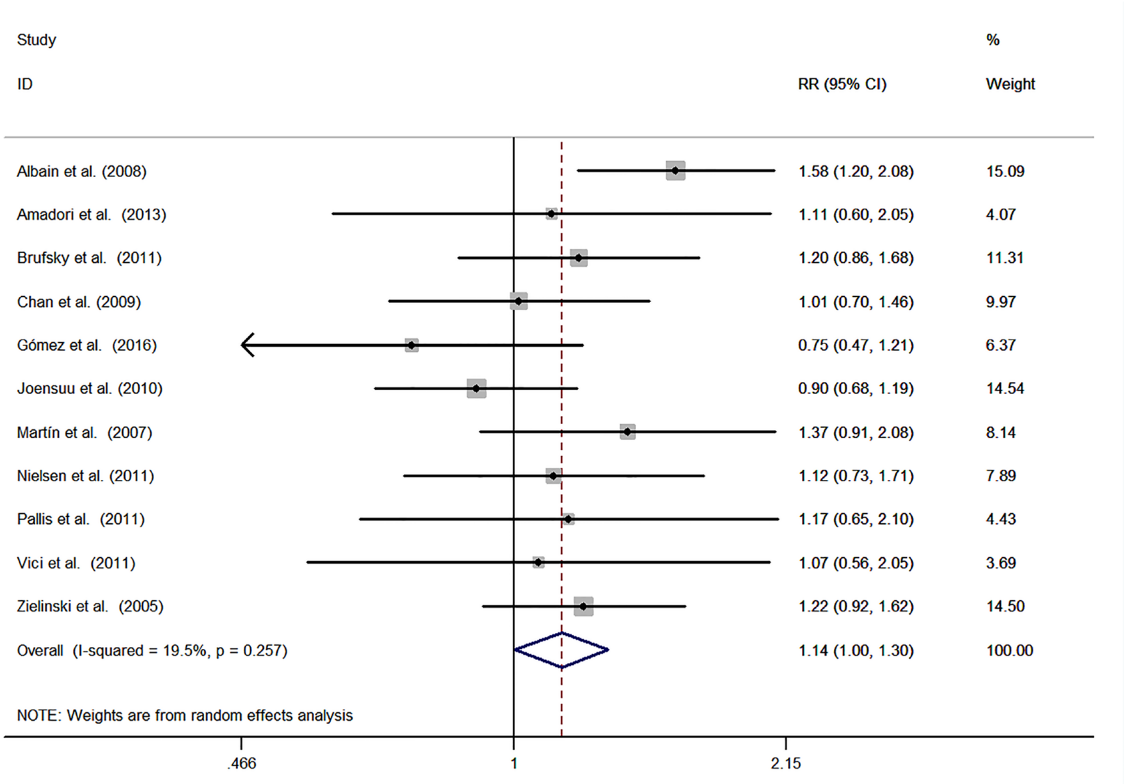


**Supplementary Figure 3: Comparisons of objective tumor response rate between 2 arms with random-model.**

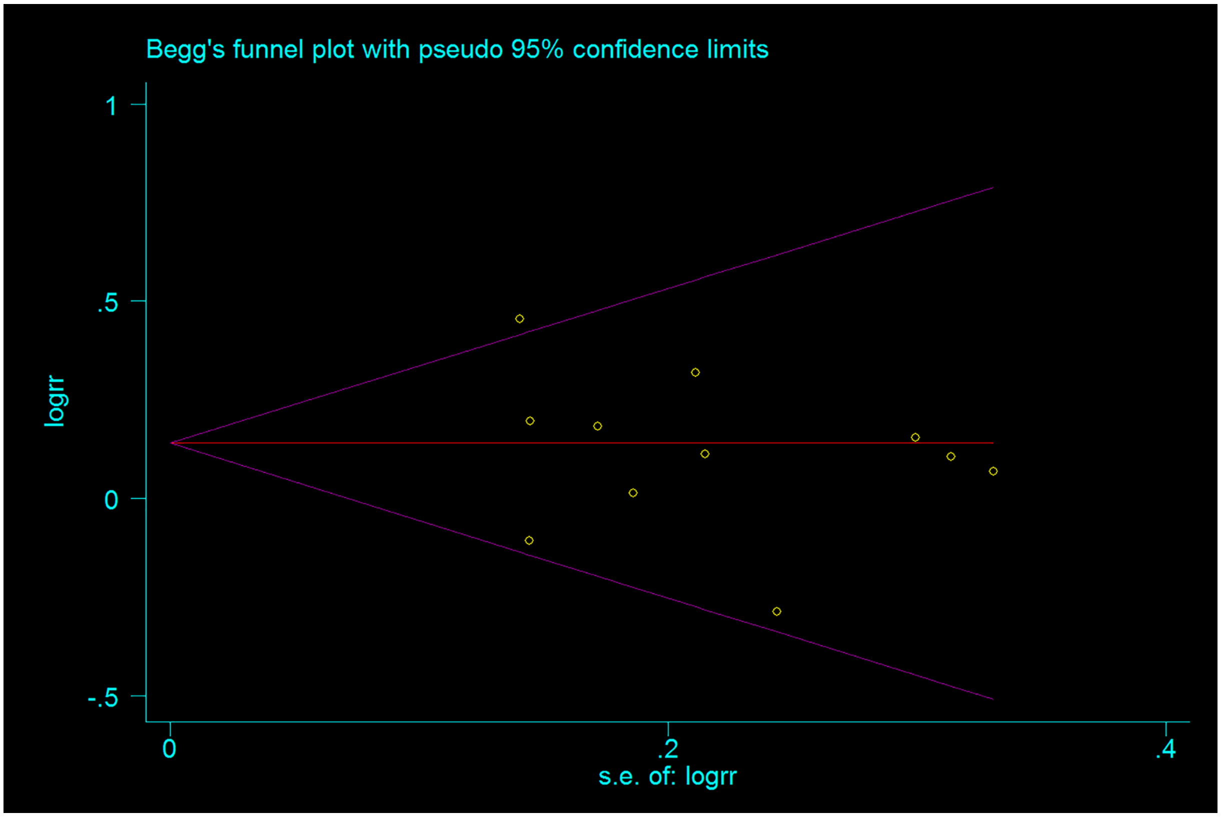


**Supplementary Figure 4: Funnel plots of objective tumor response rate between patients with gemcitabine-containing regimens and patients with non- gemcitabine-containing regimens (Egger's tests).**
